# A network approach to discerning the identities of *C. elegans* in a free moving population

**DOI:** 10.1038/srep34859

**Published:** 2016-10-11

**Authors:** Peter B. Winter, Renee M. Brielmann, Nicholas P. Timkovich, Helio T. Navarro, Andreia Teixeira-Castro, Richard I. Morimoto, Luis A. N. Amaral

**Affiliations:** 1 Department of Chemical and Biological Engineering, Northwestern University, Evanston, IL, USA; 2 Department of Molecular Biosciences, Rice Institute for Biomedical Sciences, Northwestern University, Evanston, IL, USA; 3 Life and Health Sciences Research Institute (ICVS), School of Health Sciences, University of Minho, Braga, Portugal; ICVS/3B’s - PT Government Associate Laboratory, Braga/Guimarães, Portugal; 4 Department of Physics and Astronomy, Northwestern University, Evanston, IL, USA; 5 Northwestern Institute on Complex Systems and Data Science, Northwestern University, Evanston, IL, USA; 6 Howard Hughes Medical Institute, Northwestern University, Evanston, IL, USA

## Abstract

The study of *C. elegans* has led to ground-breaking discoveries in gene-function, neuronal circuits, and physiological responses. Subtle behavioral phenotypes, however, are often difficult to measure reproducibly. We have developed an experimental and computational infrastructure to simultaneously record and analyze the physical characteristics, movement, and social behaviors of dozens of interacting free-moving nematodes. Our algorithm implements a directed acyclic network that reconstructs the complex behavioral trajectories generated by individual *C. elegans* in a free moving population by chaining hundreds to thousands of short tracks into long contiguous trails. This technique allows for the high-throughput quantification of behavioral characteristics that require long-term observation of individual animals. The graphical interface we developed will enable researchers to uncover, in a reproducible manner, subtle time-dependent behavioral phenotypes that will allow dissection of the molecular mechanisms that give rise to organism-level behavior.

A major challenge of translational biological research is to discover how molecular, cellular, and tissue level mechanisms give rise to organismal behavior. In humans, changes in behavior, such as movement, can reveal age- and disease-associated decline commonly associated with healthspan^1^. Model organisms such as nematodes^2^, flies^3^, zebrafish^4^, and mice have yielded tremendous insights into relevant cellular and molecular-level events and their relationship to physiological processes. However, behavioral data can be time-consuming to collect, is highly variable across individuals, is subject to a wide range of relevant time-scales, and can be difficult to reproduce. Our ability to relate unconstrained behavioral phenotypes to underlying biological processes is limited by our tools for acquiring large numbers of high-resolution recordings over long periods of time. Increasing the reliability and resolution of phenotypic characterization will ultimately result in a greater understanding of the molecular processes inside an organism.

The nematode *Caenorhabditis elegans* is an ideal model organism for integrating molecular information with complex phenotypes: we can control its environment and a wealth of molecular, genetic and genomic, and tissue-level information is available to contextualize healthspan^5^,^6^,^7^. Movement analysis in *C. elegans* has already been utilized to discover and map many of the neuronal and genetic components in pathways related to environmental stimulus and response, as well as innate behaviors^8^,^9^,^10^,^11^. Individual animals have been shown to exhibit a high degree of variation in movement-related behaviors that range in duration from fractions of a second^12^, to minutes^13^, hours^14^, days^15^, or weeks^16^. Quantifying how individuals differ from one another during long-term behavioral changes is critical for quantifying healthspan, yet it remains difficult to perform using current tools.

A diverse set of manual scoring^6^ and computer vision tools including single worm trackers^17^,^18^,^19^,^20^ and multi-worm trackers^21^,^22^,^23^,^24^ have been developed to quantify all aspects of *C. elegans* behavior and motility. Single-worm trackers are capable of following an individual for an extended period of time and are well suited for quantification of specific movement phenotypes such as switching between behavioral states^13^,^25^, changes in body posture^18^,^26^, or the frequency of reorientation events^27^. However, when tracking a single worm, there is a trade-off between the length of time each individual is tracked and the number of individuals that can be feasibly quantified. Researchers must, therefore, choose between long recordings of individuals to capture slow behavioral changes or recording shorter tracks for larger numbers of animals.

Because of this trade-off, a tracking program that follows many animals at once seems to be a natural choice for tracking individual differences over long periods of time. Increases in throughput, however, often come at a price: lifespan can be monitored by sacrificing the time-resolution required to follow an animal’s trajectory^16^, trajectories can be captured by sacrificing the resolution to track the posture of animals^24^, and body-postures can be captured by constraining motion of animals in a microfluidics environment^28^. Of the diverse set of multi-worm trackers, we employed the Multi-Worm Tracker (MWT)^21^ because it can capture both the trajectories and body-postures of tens of animals in an unconstrained environment. Furthermore, by tracking animals in real-time it can capture recordings using a sub-second frame rate that lasts up to a day. A primary weakness of MWT, however, is an inability to sustain the identities of animals through collisions and imaging errors. This prohibits the analysis of communication between individuals, prevents the detection of persistent individual differences, and reduces the accuracy with which each animal can be characterized. This problem, however, is not only widespread among multi-worm trackers but is also common when visually tracking larger animals^29^. However, we are unable to apply conventional methods to identify individuals using visual features because real-time tracking does not store video footage.

The two major challenges in the simultaneous tracking of multiple *C. elegans* for long periods of time are: (i) accurately resolving animals from the background, and (ii) maintaining the identity of animals as they move and collide. Resolving the former is almost always performed with segmentation, a common computer vision technique that defines clusters of adjacent pixels, typically denoted as ‘blobs’, that can be organized sequentially to form ‘tracks’.

Segmentation, however, is vulnerable to numerous conditions, including variations in lighting and the spurious background features present while tracking *C. elegans* on a bacterial lawn (Fig. 1b). Segmentation is a widespread problem and several advanced imaging techniques have been developed for correcting shape accuracy from images of worms in complex environments^30^. Limitations in segmentation can cause multiple types of errors including false positives, false negatives, and distorted shapes (Fig. 1c). All of these errors increase the difficulty in maintaining animal identity over the course of a long recording.

The greatest difficulty in retaining an animal’s identity results from direct physical interactions or collisions among animals. For example, in an experiment following 10 animals for three hours, the Multi-Worm Tracker^21^ identifies 1,750 separate tracks of which only 15% of the tracked objects move a distance greater than a single body length. To maintain identity over time we must simultaneously solve both the segmentation and interaction problems. The multiplicity of tracks for a single worm makes it impossible to identify how an animal’s behavior changes over time. The correct assignment of which tracks belong to a single worm as it moves among others would therefore resolve this dilemma. The ability to rapidly collect long, high-resolution recordings for many individuals would enable the reproducible detection of subtle behavioral phenotypes.

Here, we describe the Worm Analysis and Live Detailed Observation (WALDO) algorithm that maintains animal identity while tracking tens of animals. Furthermore, the algorithm is robust to adverse conditions with significant segmentation errors, conspecific interaction, and is compatible with real-time tracking output that lacks any pixel intensity information that would allow for the visual recognition of individual animals. WALDO implements a novel approach based on the simplification of complex networks^31^,^32^, which allows tracks that were generated by the MWT software to be sequentially ordered and related to one or more subsequent tracks that could have been created by the same individual. This mathematical representation captures the ambiguities present in the raw data and provides a framework to apply heuristic rules to reconstruct trajectories and interactions of large numbers of visually indistinguishable organisms.

## Results and Discussion

The MWT software identifies hundreds to thousands of tracks for each active worm over each hour of recording. In order to assign tracks to the same individual, we define a directed acyclic network representing all physically possible ways the individuals under study could have created the individual tracks. In our network representation, a node represents a given track and each arc (or directed edge) shows that a track could follow another in time and space, meaning that they could be from the same individual.

As our results demonstrate, this framework is flexible enough to account for a wide variety of identification errors and allows us to execute several rounds of heuristic corrections that assign tracks to specific individuals and simplify the total network structure.

### Problematic True Positives: Shape Identification

Segmentation can misidentify enough pixels that a single worm gives rise to two separate blobs (Fig. 2a). We define two operations – consolidation and pruning – that reverse this class of errors. Pruning removes any parentless or childless nodes that are tracked for less than one second. This issue arises when the split segments are momentarily lost (Fig. 2b). Consolidation combines all nodes from a specific network motif that spans less than three seconds and is composed of a parent node connected to multiple intermediary nodes that eventually all connect to another single child node. This issue comes into play when an animal is split into multiple blobs whose tracks eventually converge (Fig. 2b). Together, consolidation and pruning are responsible for half of the network simplification operations implemented by WALDO (see Supplementary Table 4).

### Problematic True Positives: Collisions

When two animals are in close physical proximity, they can be mis-identified as a single blob that persists until they achieve substantial physical separation. Figure 2a illustrates how a collision between two worms produces a sub-graph comprising 5 nodes and 4 arcs. In order to untangle worm identities during two-worm collisions, WALDO counts the total number of overlapping pixels between the last blobs in the pre-collision tracks and the first blobs in the post-collision tracks. The overlapping pixels are summed for both possible outcomes and an outcome is selected only if it is better than the other outcome by a given threshold value (Fig. 2b).

Our test set indicates this method is more than 99% accurate over a range of different parameter values (Supplementary Fig. 2). The accuracy is achieved by avoiding difficult interactions. No solution will be selected if two worms partially trade places and both outcomes have nearly equal amounts of overlap. Nor will a solution be attempted if two worms move together away from their starting positions. In total, no solution was provided for 32% of the detected collisions in our validation screen (Supplementary Table 2). As a result, recordings with high densities of interacting worms may still contain a relatively large degree of track fragmentation after this procedure.

### False negatives: Inferring Missing Arcs

The MWT can momentarily stop tracking a worm when: (a) the worm crosses a portion of the field of view with poor contrast; (b) if a frame was dropped during real-time tracking; (c) if the worm touches the edge of the image; or (d) if the worm body is split into multiple blobs that are not identified as being possibly connected. All of these computer vision errors give rise to nodes that are sources, sinks, or isolated nodes. Regardless, to maintain the identity of an animal during the recording, we need to be able to connect sinks and sources that may correspond to the same animal (Fig. 2b). Thus, in order to discover arcs that might have been missed during data collection, we examine every potential pairing involving a sink and source node and estimate whether it is plausible that the same animal gave rise to these nodes.

A missing arc is added for connections with small time and distance gaps (Δt and Δd). Arcs are added to the network only if the distance gap is smaller than ~1 body-length (50 pixels) and the time gap is less than 10 seconds (50 frames). If multiple options are available, only the connection with the smallest Δt × Δd (frames x pixels) is selected. In our validation screen, this threshold was shown to find 86% of the missing arcs between tracks created by the same animal without introducing false positives (Supplementary Table 3).

### Implementing Multiple Operations

The animal identity assigned to a track after implementing a set of operations can depend on the order in which the operations are performed. The order of operations is particularly important in a subset of sub-graphs that contain overlapping motifs (Supplementary Fig. 1a). Our analysis reveals that the sequence of operations that yields best results is: i) identify and untangle collision nodes, ii) infer gaps, iii) prune tracks, and iv) consolidate tracks. In Supplementary Fig. 1b, we illustrate how network simplification iteratively merges tracks that belong to the same individual.

In the ideal case, all tracks for the same animal will be merged into one node that is isolated from the rest of the network. Using MWT to track 10 animals, none of the animals are tracked for over 50% of the recording’s duration. With WALDO, 41% of tracks were longer than 90% of the recording, and 26% of the animals are tracked for over 99%.

#### Track Identity and Accuracy Validation

We visually screened 300 tracks created by WALDO from nine recordings to identify and quantify tracking errors and undesirable properties (See Methods). Three types of errors were discovered: 1.3% of tracks incorrectly switched between worms, 6% of tracks contained undetected collisions with a second worm, and 6.7% of the final tracks were collisions of two or more animals (Supplementary Table 1). Both the id-switch errors and the undetected collisions were caused by situations not explicitly programed into the algorithm such as three-worm collisions or contact with untracked worms. Unlike id-switches, the failure to recognize a collision includes segments of track that inaccurately represent the animal’s shape, but do not falsely change the identity of the animal. The longest observed instance of this error lasted for 69 seconds; however, the median disruption is less than 9 seconds. The inclusion of ‘collision tracks’, which record the combined shape of two or more worms, can similarly introduce noise into an analysis. However, 90% of collisions last for less than 45 seconds. By removing tracks that last less than 1 minute, only 0.8% of remaining tracks follow multi-animal collisions.

### What We Can Learn With Longer Tracks For More Animals

*C. elegans* can exhibit different types of behaviors and behavioral changes across a variety of conditions, stimuli, and time scales. As a result, researchers have used diverse experimental protocols based on their constraints and goals that vary in how quickly individuals can be assessed, the speed at which experimental conditions can be tested, the resolution of animal’s body posture, the environmental control and what type of stimulus can be delivered. Consequently, the methodology employed among representative papers (Fig. 3a) varies widely when comparing the acclimation and observation times (for sources, see Supplementary Table 5). Many of the protocols observe motility for less than a minute. Furthermore, the papers that have longer recordings sometimes use non-overlapping observation periods.

### Longer Observation Periods Provide Context

As an example of a dynamic change in behavior observed over the course of several hours, we shifted animals from their growth condition (20 °C) to a new lower or higher temperature, mechanically tapped to stimulate movement, and recorded. The worms shifted to 25 °C show a much higher initial movement rate that decays quickly, whereas animals shifted down to 15 °C exhibit an initial slower motility but remain at the same level for nearly two hours (Fig. 3b). As a result, there is a period in which 25 °C worms move faster, a period in which 15 °C worms move faster and two periods in which there is no discernable difference between the two conditions. Thus, in conditions where animals are changing behavior slowly, the seemingly inconsequential choice of the observation period can dramatically alter the outcome. The accessibility of long recordings is therefore crucial to obtain robust, reproducible phenotypes.

### Characterizing Behavioral Consistency requires Large Numbers of Individuals

Individuals in a homogeneous population of worms often show large behavioral variability. For example, the three animals whose data is shown in Fig. 4a show considerable variation in how long they remain agitated after exposure to a tapping stimulus and how often they switch back and forth between roaming and dwelling states. Quantifying how prevalent these behaviors are within a population requires capturing long tracks for a sufficiently large number of individuals.

The data displayed in Fig. 4b demonstrates that individuals within a population can be classified into multiple subcategories. We classify worms as either active or inactive during different observations periods. At 20 °C, we find that 80% of individuals are active during the first 30 minutes, but that only about 30% of individuals remain active at later times (Fig. 4c). Intriguingly, data collected for populations of worms studied at 15 °C and 25 °C have different distributions of activity levels with time. When contrasting two or more experimental conditions with a sufficiently large number of long-observations for each individuals, we can begin to probe whether or not worms in each condition exhibit the same set of behaviors. While we demonstrate this capability using a very simple metric of activity, the MWT based data provides a variety of activity, posture, and trajectory based attributes that can be quantified^21^.

## Summary

**Figure 1:**
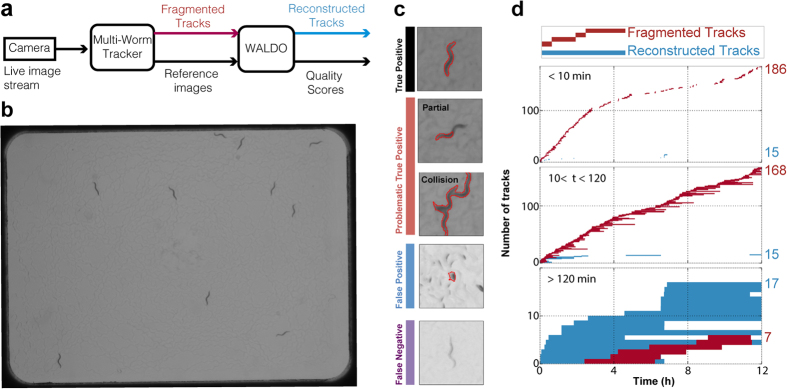
WALDO combines tracks that have been disrupted by collisions and segmentation errors. (**a**) A schematic showing how WALDO extends the multi-worm tracker’s functionalities. (**b**) Ten day-2 adult worms on a bacterial lawn that are being tracked for 12 hours. (**c**) A set of images representing correct tracking and several classes of disruptions that can interfere with maintaining animal identity. (**d**) A plot showing when and how long each blob is actively being tracked before the identity of the individual is lost. The red and blue bars show before and after WALDO is used to reconstruct an individual’s track.

WALDO is an open-access network that builds on the multi-worm tracker to provide long-term movement analysis of individual *C. elegans* nematodes while maintaining the identity of each animal in a free moving population. The ability of WALDO to disambiguate the multitude of tracks generated on an agar plate containing up to 60 adult animals over one day required the implementation of a directed acyclic network to convert thousands of short tracks into long contiguous trails. To demonstrate the performance of the WALDO algorithm, we analyzed 83 recordings monitoring a range of 10 to 60 worms. On average, WALDO corrected 3,300 disruptions per three-hour recording of 10 animals (see Supplementary Table 4). This enabled the tracking of 41% of the population for over 90% of the recording period. 25% of the population could be followed for 99% of the observation period (see example Fig. 1e). Only 1.6% of manually scored tracks contain id-switches between animals (Supplementary Table 1). Certain measurements, such as the calculating the frequencies of rare events or computing fractal properties, require long-time series following a single individual. The capability of analyzing more individuals for long periods of time significantly expands the types of properties that can be readily acquired with multi-worm tracking software.

We demonstrated the capability of WALDO to reveal unpredicted behaviors that could cause experimental inconsistencies with shorter observation periods by following individual animals for long periods of time under different ambient temperatures (15, 20, 25 °C) (Figs 3b and 4). Each ambient condition affects, in a non-monotonic manner, a population of worms’ relaxation time after a perturbation to stimulate movement (Fig. 3b). Moreover, individuals within each population exhibit a wide range of behavioral differences that cause large measurement fluctuations in small samples and can only be classified and quantified by following a sufficiently large number of individuals over the course of several hours (Fig. 4). WALDO, therefore, provides a new methodological complement for animal behavior tracking analysis by efficiently quantifying behavioral changes that require recording over periods and assessing large numbers of individuals.

## Methods

### *C. elegans* strain and culturing

All assays were conducted with the wild-type Bristol isolate of *Caenorhabditis elegans* (N2), obtained from the *Caenorhabditis* Genomic Center (CGC). Standard methods were used for culturing and observing C. *elegans*^33^. Nematodes were grown at 20 °C on 60 mm nematode growth medium (NGM) plates seeded with *Escherichia coli* OP50 strain. To obtain the age synchronized population of eggs, gravid adults were allowed to lay eggs for 30 min on OP50 plates and were then removed. The eggs were allowed to hatch and develop into young adults (day 1 of adulthood) at 20 °C.

### Motility Experiments

All recordings were performed inside of a Percival I-36NL C8 incubator to ensure a constant temperature environment. Recordings were captured using three Dalsa Falcon 4M30 (monochrome, 4 megapixel, 30 frames per second) cameras with Rodenstock 60 mm f/4.0 enlarging lenses connected to Dell Optiplex 790 (Intel i5-2400, 4 GB RAM) computers with a National Instruments Camera Link card running the Multi-Worm Tracker as described previously^21^. The Multi-Worm Tracker settings used were: 7% object Contrast, 50% fill hysteresis, 10% object size hysteresis, 1 Image Binning, 10 Image Adaptation Bands, an Adaptation Rate of 5, 100 frames of Adaptation, and an object border of 20. We also used Using Dark Objects, Skeletonize, Contour, Aggregate Output, and the Bit Depth from the Camera. A raw image was saved every one to five minutes for regular recordings. For recordings explicitly used for validation, an image was saved every second. Collisions with 100 or more pixels of overlap were resolved. Missing arcs were inferred if Δt was smaller than 50 frames and Δd was smaller than 50 pixels.

Recordings were performed on 60 mm NGM plates seeded with 200 μL of OP50 bacteria, covering the entire surface of the agar. A custom-made copper frame with 2.5 × 1.5 cm interior dimensions was placed onto the bacteria in order to prevent worms from leaving the field of view. Ten to sixty day-1 adult animals were transferred onto the bacterial lawn inside the copper frame. The plates were moved into the recording incubator and allowed to acclimate to the interior temperature for 30–60 minutes to prevent condensation on the plate lid. Before recordings began, plates were manually tapped to stimulate movement and recordings were started within 30 seconds.

For temperature-shift experiments, worms were raised to day-1 adulthood and transferred to recording plates with OP50 as previously described. The recording plates were placed into an incubator set to either 15, 20, or 25 °C. Plates were allowed to acclimate to the incubator’s temperature for 30 minutes before they were tapped and recorded for three hours.

### Centroid Speed Calculations

WALDO stitches together tracks from multiple track fragments, which often leaves multiple gaps in observing an animal’s trajectory. While performing analysis of centroid speeds, we linearly interpolate centroid positions for missing sections less than 1 second as the worm’s position remains relatively constant over such a short time. Missing sections longer than one second were excluded from analysis. To reduce jitter in worm position, the x and y coordinates for each continuous track portion were smoothed using a one second running average. Because our analysis focuses on long-term changes in activity level, we smoothed the speed measurements using a one-minute running average. To create the aggregated speed profiles for all worms on one or more plates, worm speeds from all individuals were binned on one-minute intervals and the mean from each bin was plotted.

### Identifying Collisions

**Figure 2:**
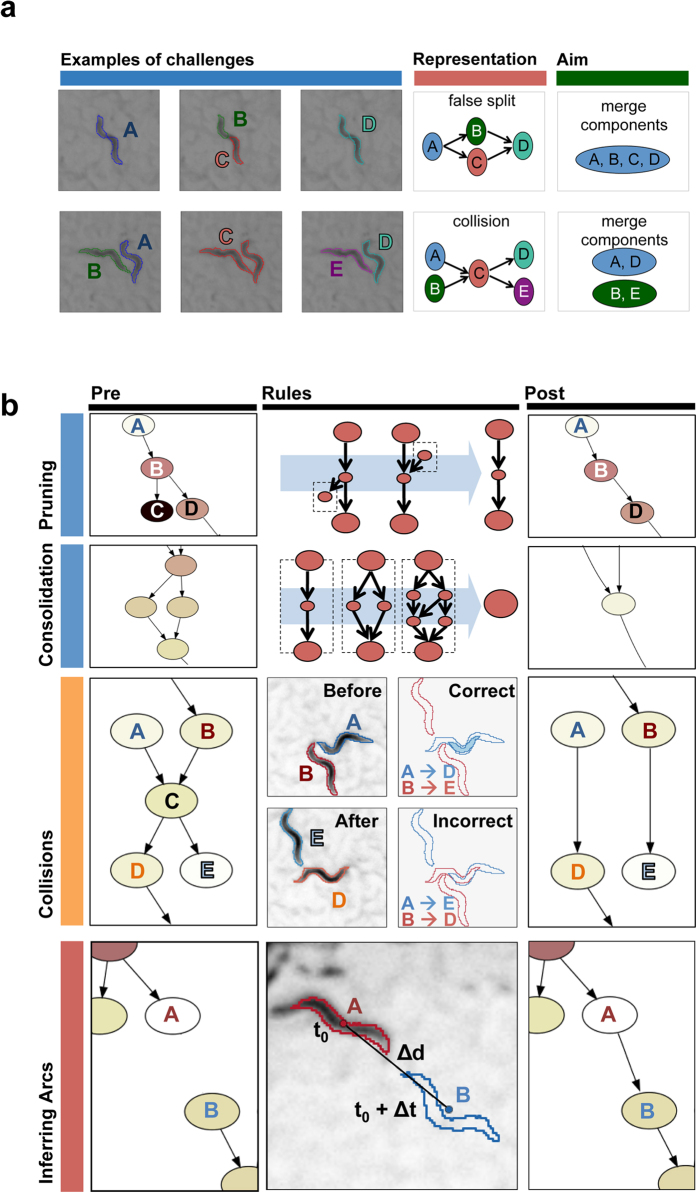
A directed acyclic network provides a means to organize problems that arise in tracking multiple animals and to apply solutions. (**a**) The sequence of events in a tracking disruption can be represented as nodes connected by arcs. The first and second rows illustrate when an animal is fragmented into two blobs and when two animals come into contact with one another. (**b**) Each class of tracking disruption can be corrected using a different operation. Pruning and consolidation removes track fragments that were created when a single worm was incorrectly split into multiple blobs. When a collision occurs, the identities of animals are calculated using the amount of overlapping pixels of each blob pair before and after the collision. To discover connections between tracks that were not included in the tracking data, missing arcs are inferred using position and time deltas.

**Figure 3:**
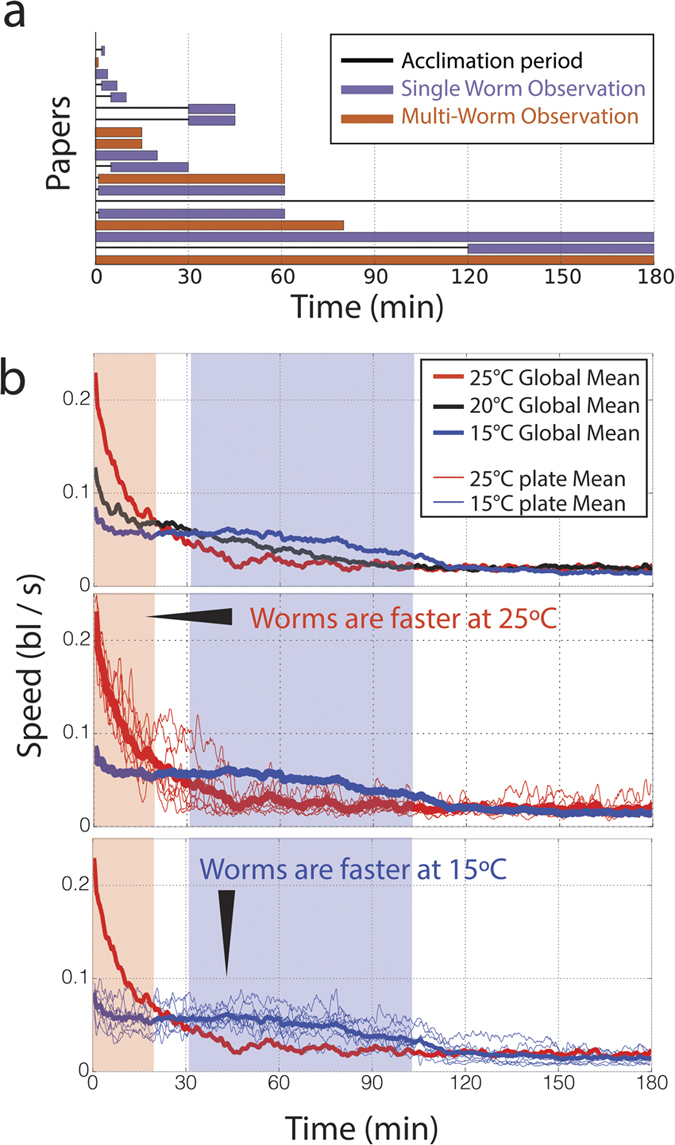
Long observations are required to discover slow changes in behavior. (**a**) Reported observation and acclimation periods vary dramatically from paper to paper. Each row represents a *C. elegans* motility experiment reported in a paper^13^,^15^,^18^,^24^,^34^,^35^,^36^,^37^,^38^,^39^,^40^,^41^,^42^,^43^,^44^,^45^,^46^,^47^. The shaded region indicates the period in which animals were actively observed. The line at the beginning indicates the acclimation period before animals measurements are acquired. (**b**) Shows the average speed across time of worms raised at 20 °C until early adulthood whereupon they were shifted to 15 or 25 °C and subjected to mechanical stimulation. These plots show the first three hours immediately following the mechanical tapping. Bold lines indicate aggregated averages of all animals at a given temperature. Smaller lines indicate averages for a plate of 10 animals.

**Figure 4:**
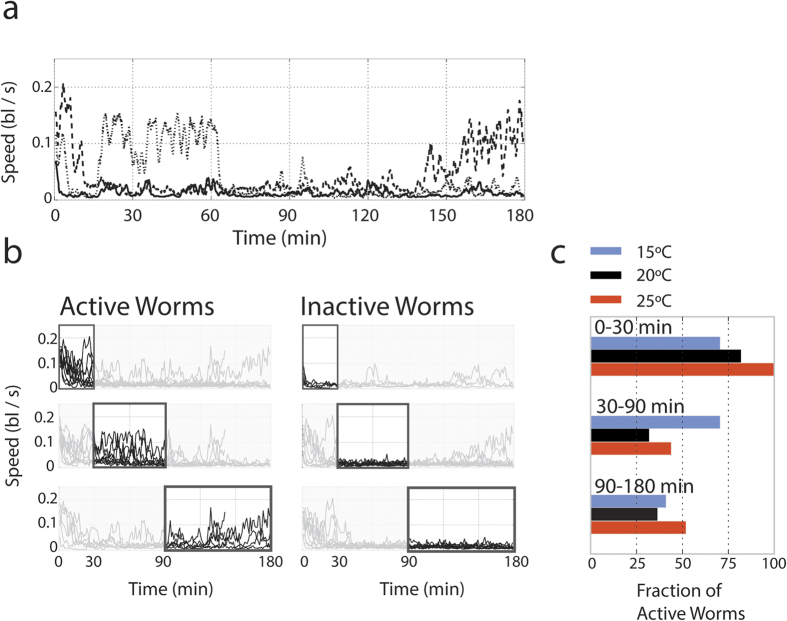
A large enough set of behavior profiles from individual animals allows populations of *C. elegans* to be classified into behavioral subgroups. **(a)** Three individuals show diverging behaviors despite originating from the same genetic background (N2) and being recorded under the same conditions (day-1 adult, hermaphrodites recorded on OP50, at 20 °C). **(b)** A rudimentary approach for finding distinct types of behaviors within the same population is to divide the individuals based on their levels of activity. The groupings differentiate active and inactive animals from the 20 °C experimental condition (shown in Fig. 3) by classifying them as having more or less than 5 min of active movement in the first 30 minutes, the middle 30 to 90 minutes and the final 90 to 180 minutes of the recording. **(c)** Comparing the prevalence of different behaviors across the temperature conditions used in Fig. 3.

This network motif in Fig. 2a is not unique to collisions and can arise from other processes and errors in track creation. Further profiling of actual collisions reveals that frequently, at least one of the worms in a collision crawls for a distance greater than 1 body-length distance before or after making contact with another worm. We use this observation as our primary means to identify collisions and flag any blobs that match this criterion and occupy the ‘node c’ position in the collision motif.

### Validation of WALDO Operations

315 tracks from nine recordings were scored independently by two researchers to check for errors in maintaining individual identity. Each recording tracked 12 to 30 worms for 10-minutes. Images were saved using MWT at 1 Hz. Missing arcs were inferred with a Δt and Δd of 50 frames, and 50 pixels. An overlap threshold of 100 pixels was used to resolve collisions. The centroids and id of each track was drawn onto the images so that researchers could step through images in order to follow how each animal moved across the plate and interacted with other worms. This method allowed screeners to validate whether tracks contained id-switches, undetected collisions, or followed multiple animals. The screeners agreed with one another on 97% of the reviewed tracks. Tracks where the screeners disagreed were reviewed again to establish consensus. The results of track identities, collision resolution and inferring missing arcs are reported in Supplementary Tables 1, 2 and 3.

### Literature Survey

Papers were included in the literature search if they met the following three criteria: (1) a motility assay was performed using crawling worms, (2) software was used to track some aspect of the animal’s behavior, and (3) the duration of observation was reported in the text of the paper. We reported the acclimation time worms have without any stimulus before they are observed and how long the animals were observed for. A full table of the findings is included in Supplementary Table 6.

### Distribution of Software and Source Code

We are releasing the source code for WALDO under the MIT open source license and the code repository is freely available for download (https://github.com/amarallab/waldo). A compiled version of WALDO for Windows 8 is included in Supplementary Software. Newer versions of the user manual and compiled code will be made available as well (http://amaral-lab.org/resources/software/waldo).

## Additional Information

**How to cite this article**: Winter, P. B. *et al*. A network approach to discerning the identities of *C. elegans* in a free moving population. *Sci. Rep.* **6**, 34859; doi: 10.1038/srep34859 (2016).

## Supplementary Material

Supplementary Information
