# Supplementary material for: A network approach to discerning the identities of C. elegans in a free moving population
Source: Sci Rep. 2016 Oct 11;6:34859. doi: 10.1038/srep34859 (PMC5057085; doi:10.1038/srep34859)
Supplement: Supplementary Information [file srep34859-s1.pdf]

A network approach to discerning the identities of *C. elegans* in a free moving population

Peter B. Winter[1,*], Renee M. Brielmann[2], Nicholas P. Timkovich[1], Helio T. Navarro[1], Andreia Teixeira-Castro[2,3], Richard I. Morimoto[2*], and Luis A. N. Amaral[1,4,5,6*]

[1]Department of Chemical and Biological Engineering, Northwestern University, Evanston, IL, USA
[2]Department of Molecular Biosciences, Rice Institute for Biomedical Sciences, Northwestern University, Evanston, IL, USA
[3]Life and Health Sciences Research Institute (ICVS), School of Health Sciences, University of Minho, Braga, Portugal; ICVS/3B's - PT Government Associate Laboratory, Braga/Guimarães, Portugal.
[4]Department of Physics and Astronomy, Northwestern University, Evanston, IL, USA
[5]Northwestern Institute on Complex Systems and Data Science, Northwestern University, Evanston, IL, USA
[6]Howard Hughes Medical Institute, Northwestern University, Evanston, IL, USA
[*]Correspondence should be addressed to Richard I. Morimoto (r-morimoto@northwestern.edu) and Luis A. N. Amaral (amaral@northwestern.edu)

recording	worms	#tracks			WALDO tracks	
id	recorded	MWT	WALDO	human	id-preserved	id-switched
0	12	55	14	13	14	0
1	17	160	24	19	24	0
2	18	239	24	19	24	0
3	18	160	22	19	22	0
4	24	505	37	30	37	0
5	24	368	38	31	37	1
6	28	611	35	28	33	2
7	30	357	48	31	47	1
8	30	783	58	40	58	0
totals	201	3238	300	230	296	4

Supplementary Table 1: Track-Joining Completeness and ID-Integrity. This table shows an overview of the track validation screen (see methods). The *worms recorded* column indicates the number of animals in the image frame at t=0. The next three columns show that MWT, WALDO and Human indicate the number of created by the Multi-Worm Tracker, WALDO, and by manual curation, respectively. Tracks from manual curation were based on WALDO's output but include additional solutions for collisions between animals and connect more nodes with additional missing arcs. The id-preserved and id-switched numbers show how many of WALDO's tracks correctly follow the same individual or incorrectly switch individuals.

recording	worms	collisions			id-switches	
id	recorded	total	resolved	unresolved	collision	other
0	12	13	8	5	0	0
1	17	32	21	11	0	0
2	18	50	35	15	0	0
3	18	33	24	9	0	0
4	24	106	72	34	0	0
5	24	47	26	21	0	1
6	28	89	61	28	0	1
7	30	66	48	18	0	1
8	30	99	69	30	0	0
totals	201	535	364	171	0	3

Supplementary Table 2: Validation Screen Collisions. This table shows an overview of the collisions and events in the validation screen (see methods). The *total* column shows how many collisions WALDO detected in MWT's output. The *resolved* column indicates how many collisions WALDO solved. The *unresolved* column shows how many collisions WALDO detected but did not attempt to solve. The *id-switches* columns in this table indicate the number of events that lead to id-switches, as opposed to the amount tracks containing id-switches recorded in Table S1.

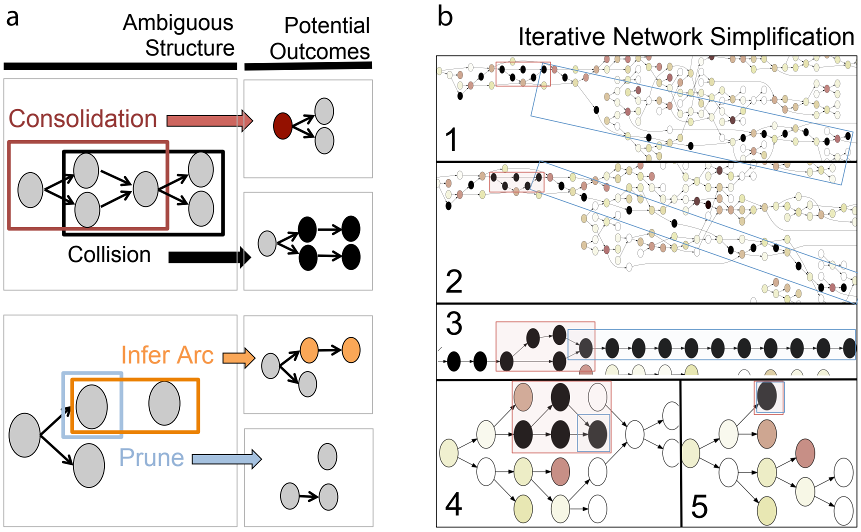

Supplementary Figure 1: a) The order in which operations are performed can influence how tracks are reconstructed. The first example shows how a consolidation could compete with a collision. The second example shows how inferring arcs can compete with pruning. b) Iterative network operations increase the duration in which each individual is followed and reduce the complexity of the entire network of track fragments. All nodes for the same individual are colored in black. The shrinking red and blue boxes show how two groups of nodes are combined through successive rounds of simplification. The darker the color, the longer the track it represents.

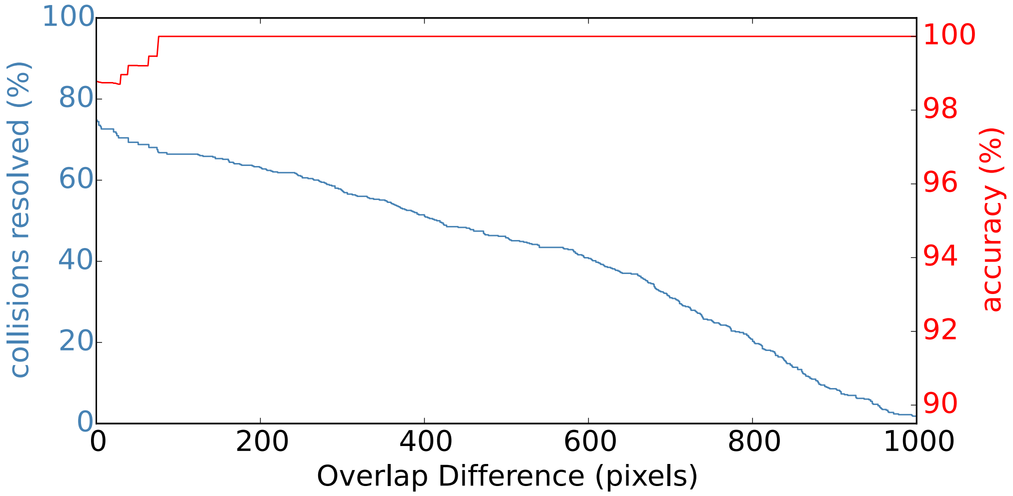

Supplementary Figure 2: Parameter Values for Collision Resolution. *Overlap difference* is the only parameter WALDO uses when resolving collisions: the minimum difference between overlapping pixels for each possible outcome (See Fig 2.b). Here we show the percent of the 535 collisions detected in the validation screen that are resolved with a given threshold (blue) and the percent of collisions that are resolved correctly (red). Worms sizes used in this screen ranged from 700 to 950 pixels. The accuracy improves for higher overlap differences, however, percent of collisions declines. All recordings were analyzed with an overlap difference of 10 or 100 pixels.

recording	worms	missing arcs inferred			
id	recorded	total	joined	missed	incorrect
0	12	1	1	0	0
1	18	4	3	1	0
2	18	1	1	0	0
3	24	2	2	0	0
4	24	8	8	0	0
5	28	5	5	0	0
6	30	4	2	2	0
7	30	6	4	2	0
totals	184	31	26	5	0

Supplementary Table 3: Validating Missing Arcs Inference. This table shows an overview of the missing arcs detected in the validation screen (see methods). An inferred arc was marked as *joined* if it was included in a WALDO track. A track was marked as *incorrect* if it was included in WALDO track and the screener could detect that it was not the same individual. An arc was scored as *missed* if an animal's track was discontinued and the screener was able to follow the same individual until it was once again assigned to a new track.

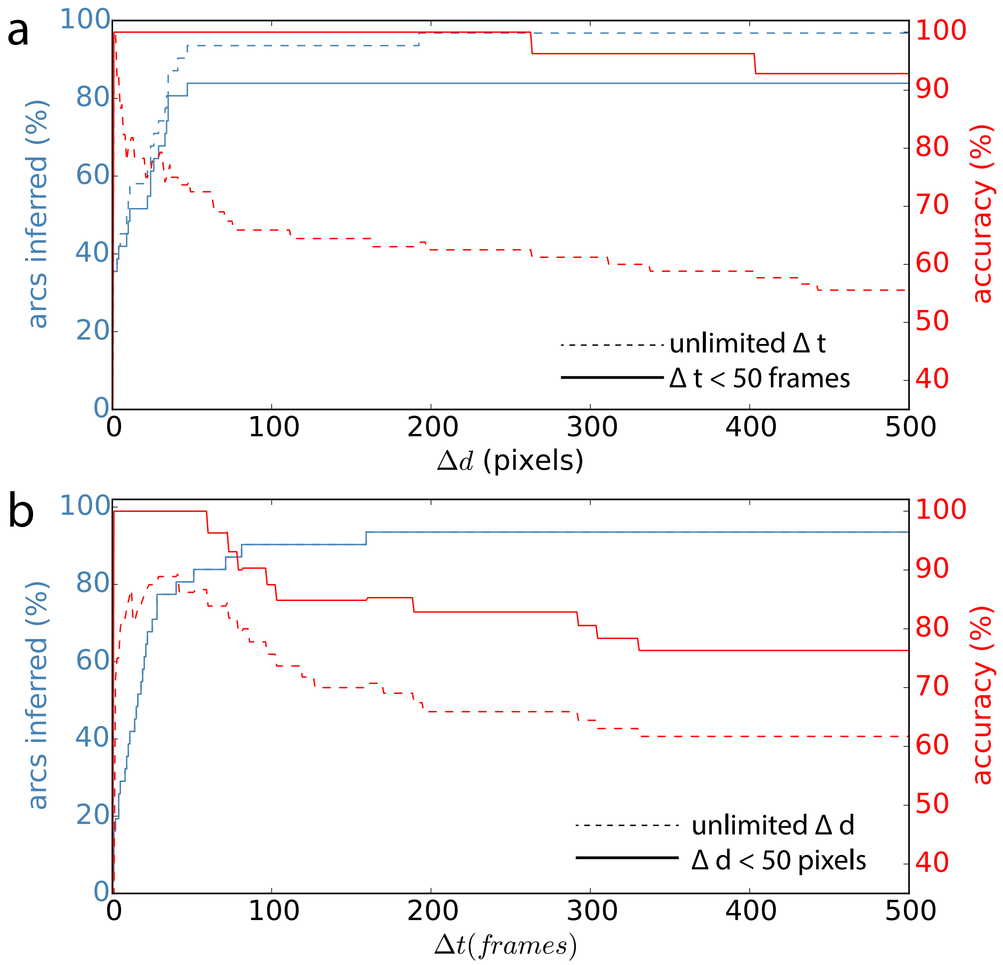

Supplementary Figure 3: Parameter Values for Inferring Arcs. Missing arcs are inferred if the start of one track occurs within a fixed time and distance of the termination a previous track. The two parameters in this algorithm are Δt and Δd, the time and distance thresholds. Blue lines indicate the percent of all true missing arcs found. Red lines indicate the percent of arcs that correctly join tracks that belong to the same individual. Dashed lines indicate the results that would be obtained if the other parameter were not considered. Solid lines are obtained if one parameter is held constant at the value we use to analyze recordings.

Supplementary Table 4: Network operations performed on recordings.

id	total minutes	#worms	MWT tracks	WALDO tracks	difference	prune	consolidate	resolve collisions	infer gaps
20150504_123807	180	5	698	13	291	0	183	108	0
20150505_111654	180	10	677	13	483	1	233	243	6
20150505_150046	180	10	1964	51	1499	6	830	624	39
20150506_115813	180	10	3175	204	2833	64	1606	1143	20
20150506_153738	180	5	579	24	447	15	175	255	2
20150507_103013	180	10	2367	67	2278	8	1231	1032	7
20150507_144556	180	5	411	10	389	5	128	255	1
20150508_105539	180	10	990	39	949	5	399	531	14
20150511_121312	180	10	1123	101	899	0	452	402	45
20150511_121317	180	10	1075	27	812	0	486	324	2
20150511_121322	180	10	1334	35	1289	0	721	567	1
20150511_160114	180	30	8650	257	8393	9	4426	3957	1
20150511_160118	180	20	3594	104	3474	0	1877	1596	1
20150511_160123	180	10	1857	74	1763	3	1138	612	10
20150512_110456	180	10	3273	40	2216	0	1293	921	2
20150512_110519	180	10	852	34	817	0	295	522	0
20150512_110526	180	10	860	47	747	4	240	495	8
20150512_165039	180	60	33940	2653	31270	34	15609	15507	120
20150512_165044	180	50	28642	2760	25831	32	11561	14076	162
20150512_165052	180	40	13715	907	12759	19	4461	8234	45
20150513_112911	180	10	1299	38	1201	8	496	693	4
20150513_112917	180	10	1705	64	1287	2	358	924	3
20150513_144241	180	10	10611	66	1173	2	336	753	82
20150513_144253	180	10	1371	72	1230	2	497	729	2
20150513_144302	180	10	740	37	668	3	189	471	5
20150514_121513	180	10	1041	55	982	0	339	642	1
20150514_121515	180	10	1132	26	898	1	426	468	3
20150514_121528	180	10	857	50	711	2	208	489	12
20150514_164158	180	10	838	36	733	1	278	444	10
20150514_164204	180	10	1454	41	810	1	192	609	8
20150515_124728	180	10	1083	23	921	19	361	534	7
20150515_124736	180	10	1571	79	1343	27	467	837	12
20150515_124840	180	10	1181	32	1109	5	322	780	2
20150515_155539	180	10	654	18	628	0	295	330	3
20150515_155545	180	10	618	20	549	1	173	372	3
20150518_121756	180	10	1014	52	817	4	472	312	29
20150518_121757	180	10	853	32	821	0	478	342	1
20150518_121809	180	10	905	31	871	0	477	384	10
20150518_182452	180	10	473	22	448	0	195	246	7
20150518_182454	180	10	1408	33	1315	18	895	390	12
20150518_182501	180	10	1294	25	1235	1	706	519	9
20150519_122411	180	10	1761	204	1498	4	125	1356	13
20150519_122413	180	10	1159	55	1101	0	498	603	0
20150519_122420	180	10	795	23	761	0	271	489	1
20150519_170520	180	10	676	17	600	0	304	288	8
20150519_170525	180	10	1799	122	1515	11	492	999	13
20150519_170530	180	10	1576	166	1340	0	99	1227	14

table continued next page

Supplementary Table 4: Network operations performed on recordings.

id	total minutes	#worms	MWT tracks	WALDO tracks	difference	prune	consolidate	resolve collisions	infer gaps
20150608_121717	180	30	6298	446	5534	14	3226	1977	317
20150608_121725	180	10	1876	30	1828	0	1265	543	20
20150608_121812	180	50	18037	779	16677	76	10841	5220	540
20150608_165515	180	40	10764	460	10174	4	5279	4842	49
20150608_165523	180	60	23539	844	22649	5	13174	9450	20
20150608_165610	180	20	2824	93	1932	1	957	891	83
20150615_124720	180	10	666	25	603	1	276	315	11
20150615_124728	180	10	984	21	907	0	525	378	4
20150615_124738	180	10	2139	47	2053	0	1322	705	26
20150615_170747	180	10	1234	38	1192	1	679	492	20
20150615_170754	180	10	1116	22	1094	1	722	369	2
20150615_170759	180	10	661	17	644	1	307	336	0
20150622_121933	180	40	8204	232	7802	0	4006	3783	13
20150622_121935	180	20	2810	58	2709	3	1603	1092	11
20150622_121940	180	60	19103	808	18076	4	9404	8643	25
20150625_132445	180	10	1560	19	1537	1	909	621	6
20150625_132448	180	10	868	34	789	0	393	390	6
20150625_132449	180	10	1123	45	991	1	632	306	52
20150625_180232	180	10	1139	71	790	14	476	258	42
20150625_180236	180	10	852	17	835	0	448	384	3
20150625_180237	180	10	1409	24	1385	1	823	558	3
20150626_111855	180	40	19319	949	18358	14	10560	7782	2
20150626_173234	180	30	7678	205	7268	5	4584	2646	33
20150626_173245	180	50	22422	1114	19155	33	11377	7509	236
20150626_173250	180	20	3182	119	2917	14	1451	1440	12
20150629_113741	180	40	21186	878	20158	11	13598	6267	282
20150629_113757	180	20	7882	218	7648	1	5405	2232	10
20150629_113803	180	50	17689	727	16924	4	9065	7836	19
20150629_165904	180	10	464	47	381	5	172	186	18
20150629_165915	180	60	24550	1091	22813	23	11951	10734	105
20150629_165920	180	30	5396	282	4980	2	2459	2484	35
20150702_113855	180	10	13665	1909	10340	190	2818	873	6459
20150702_113859	180	10	6613	1585	4429	61	1741	351	2276
20150702_150024	180	10	1510	21	1334	0	977	354	3
20150702_150028	180	10	3573	309	3217	21	2174	864	158

Supplementary Table 4: Network operations performed on recordings. This table shows basic information about the recording as well as how many times each of the network operations (Fig. 2b) were performed while correcting each of the recordings.

Supplementary Table 5: Number of tracks

	total	#	total tracks		moving tracks		interuptions		
id	minutes	worms	MWT	WALDO	MWT	WALDO	MWT	removed	removed (%)
20150504_123807	180	5	698	13	26	5	693	685	99
20150505_111654	180	10	677	13	67	13	667	664	100
20150505_150046	180	10	1964	51	267	38	1954	1913	98
20150506_115813	180	10	3175	204	391	90	3165	2971	94
20150506_153738	180	5	579	24	93	19	574	555	97
20150507_103013	180	10	2367	67	309	46	2357	2300	98
20150507_144556	180	5	411	10	85	5	406	401	99
20150508_105539	180	10	990	39	155	25	980	951	97
20150511_121312	180	10	1123	101	167	69	1113	1022	92
20150511_121317	180	10	1075	27	93	27	1065	1048	98
20150511_121322	180	10	1334	35	143	22	1324	1299	98
20150511_160114	180	30	8650	257	847	176	8620	8393	97
20150511_160118	180	20	3594	104	374	86	3574	3490	98
20150511_160123	180	10	1857	74	167	38	1847	1783	97
20150512_110456	180	10	3273	40	173	25	3263	3233	99
20150512_110519	180	10	852	34	171	32	842	818	97
20150512_110526	180	10	860	47	195	33	850	813	96
20150512_165039	180	60	33940	2653	1387	761	33880	31287	92
20150512_165044	180	50	28642	2760	2005	895	28592	25882	91
20150512_165052	180	40	13715	907	1692	523	13675	12808	94
20150513_112911	180	10	1299	38	241	32	1289	1261	98
20150513_112917	180	10	1705	64	329	53	1695	1641	97
20150513_144241	180	10	10611	66	315	40	10601	10545	99
20150513_144253	180	10	1371	72	281	62	1361	1299	95
20150513_144302	180	10	740	37	191	29	730	703	96
20150514_121513	180	10	1041	55	265	41	1031	986	96
20150514_121515	180	10	1132	26	141	25	1122	1106	99
20150514_121528	180	10	857	50	201	26	847	807	95
20150514_164158	180	10	838	36	129	21	828	802	97
20150514_164204	180	10	1454	41	214	35	1444	1413	98
20150515_124728	180	10	1083	23	164	16	1073	1060	99
20150515_124736	180	10	1571	79	274	58	1561	1492	96
20150515_124840	180	10	1181	32	251	25	1171	1149	98
20150515_155539	180	10	654	18	120	14	644	636	99
20150515_155545	180	10	618	20	140	15	608	598	98
20150518_121756	180	10	1014	52	121	44	1004	962	96
20150518_121757	180	10	853	32	119	23	843	821	97
20150518_121809	180	10	905	31	84	22	895	874	98
20150518_182452	180	10	473	22	112	18	463	451	97
20150518_182454	180	10	1408	33	86	28	1398	1375	98
20150518_182501	180	10	1294	25	95	25	1284	1269	99
20150519_122411	180	10	1761	204	804	160	1751	1557	89
20150519_122413	180	10	1159	55	188	45	1149	1104	96
20150519_122420	180	10	795	23	162	18	785	772	98
20150519_170520	180	10	676	17	53	13	666	659	99
20150519_170525	180	10	1799	122	422	91	1789	1677	94
20150519_170530	180	10	1576	166	692	135	1566	1410	90

table continued next page

Supplementary Table 5: Number of tracks

	total	#	total tracks		moving tracks		interuptions		
id	minutes	worms	MWT	WALDO	MWT	WALDO	MWT	removed	removed (%)
20150608_121717	180	30	6298	446	330	249	6268	5852	93
20150608_121725	180	10	1876	30	94	21	1866	1846	99
20150608_121812	180	50	18037	779	864	464	17987	17258	96
20150608_165515	180	40	10764	460	832	202	10724	10304	96
20150608_165523	180	60	23539	844	1428	418	23479	22695	97
20150608_165610	180	20	2824	93	242	67	2804	2731	97
20150615_124720	180	10	666	25	105	23	656	641	98
20150615_124728	180	10	984	21	71	17	974	963	99
20150615_124738	180	10	2139	47	95	32	2129	2092	98
20150615_170747	180	10	1234	38	136	31	1224	1196	98
20150615_170754	180	10	1116	22	97	17	1106	1094	99
20150615_170759	180	10	661	17	121	12	651	644	99
20150622_121933	180	40	8204	232	765	176	8164	7972	98
20150622_121935	180	20	2810	58	255	50	2790	2752	99
20150622_121940	180	60	19103	808	1228	392	19043	18295	96
20150625_132445	180	10	1560	19	58	14	1550	1541	99
20150625_132448	180	10	868	34	100	31	858	834	97
20150625_132449	180	10	1123	45	101	31	1113	1078	97
20150625_180232	180	10	1139	71	84	23	1129	1068	95
20150625_180236	180	10	852	17	101	13	842	835	99
20150625_180237	180	10	1409	24	66	13	1399	1385	99
20150626_111855	180	40	19319	949	785	276	19279	18370	95
20150626_173234	180	30	7678	205	561	137	7648	7473	98
20150626_173245	180	50	22422	1114	1232	454	22372	21308	95
20150626_173250	180	20	3182	119	337	78	3162	3063	97
20150629_113741	180	40	21186	878	866	360	21146	20308	96
20150629_113757	180	20	7882	218	196	88	7862	7664	97
20150629_113803	180	50	17689	727	1075	314	17639	16962	96
20150629_165904	180	10	464	47	90	35	454	417	92
20150629_165915	180	60	24550	1091	2136	664	24490	23459	96
20150629_165920	180	30	5396	282	668	174	5366	5114	95
20150702_113855	180	10	13665	1909	1210	1697	13655	11756	86
20150702_113859	180	10	6613	1585	835	554	6603	5028	76
20150702_150024	180	10	1510	21	84	20	1500	1489	99
20150702_150028	180	10	3573	309	326	207	3563	3264	92

Supplementary Table 5: The number of tracks before and after running WALDO. This table shows basic information about the recording as well as how many tracks are present, how many tracks move at least one body-length, and how many times the tracking of an animal was interrupted. The number of interruptions was calculated by subtracting the number of worms present in the recording from the total number of tracks generated.

Supplementary Table 6: Published worm tracking protocols

title	single or multi	delay (min)	duration (min)	key
Undulatory Locomotion of *Caenorhabditis elegans* on Wet Surfaces	single	2	1	Shen *et al.* (2012)
A genetic screening strategy identifies novel regulators of the proteostasis network.	multi	0	1	Silva *et al.* (2011)
Systematic profiling of *Caenorhabditis elegans* locomotive behaviors reveals additional components in G-protein Gαq signaling.	single	0	4	Yu *et al.* (2013)
Bending amplitude - A new quantitative assay of *C. elegans* locomotion: Identification of phenotypes for mutants in genes encoding muscle focal adhesion components	single	2	5	Nahabedian *et al.* (2012)
Synaptic polarity of the interneuron circuit controlling *C. elegans* locomotion	single	5	5	Rakowski *et al.* (2013)
A database of *Caenorhabditis elegans* behavioral phenotypes	single	30	15	Yemini *et al.* (2013)
Dynamic encoding of perception, memory, and movement in a *C. elegans* chemotaxis circuit	multi	0	15	Lou *et al.* (2014)
Bidirectional thermotaxis in *Caenorhabditis elegans* is mediated by distinct sensorimotor strategies driven by the AFD thermosensory neurons	multi	0	15	Luo *et al.* (2014)
A dictionary of behavioral motifs reveals clusters of genes affecting *Caenorhabditis elegans* locomotion	single	30	15	Brown *et al.* (2013)
Locomotion analysis identifies roles of mechanosensory neurons in governing locomotion dynamics of *C. elegans*	single	0	20	Cohen *et al.* 2012
Experiments and theory of undulatory locomotion in a simple structured medium	single	5	25	Majmudar *et al.* (2012)
High-throughput behavioral analysis in *C. elegans*	multi	240	60	Swierczek *et al.* (2011)
Dimensionality and Dynamics in the Behavior of *C. elegans*	single	1	60	Stephens *et al.* (2008)
Controlling interneuron activity in *Caenorhabditis elegans* to evoke chemotactic behaviour	multi	1	60	Kocabas *et al.* (2012)
Mechanistic analysis of the search behaviour of *Caenorhabditis elegans*	single	1	60	Salvador *et al.* (2014)
Directional Locomotion of *C. elegans* in the Absence of External Stimuli	multi	0	80	Peliti, Chuang, and Shaham (2013)
The Geometry of Locomotive Behavioral States	single	0	240	Gallagher *et al.* (2013)
Dietary choice behavior in *Caenorhabditis elegans*.	single	120	480	Shtonda and Avery (2006)
Long-term imaging of circadian locomotor rhythms of a freely crawling *C. elegans* population	multi	0	5760	Winbush *et al.* (2015)

Supplementary Table 6: Published worm tracking protocols. This table shows which papers are included in our survey of published protocols in the same order that they occur in the figure (Fig. 3a).
